# Recurrent *AKT* mutations in human cancers: functional consequences and effects on drug sensitivity

**DOI:** 10.18632/oncotarget.6648

**Published:** 2015-12-17

**Authors:** Kyung H. Yi, Josh Lauring

**Affiliations:** ^1^ The Sidney Kimmel Comprehensive Cancer Center, The Johns Hopkins University School of Medicine, Baltimore, Maryland, USA

**Keywords:** Akt, mutation, MK-2206, personalized, cancer

## Abstract

Precision oncology trials based on tumor gene sequencing depend on robust knowledge about the phenotypic consequences of the genetic variants identified in patients' tumors. Mutations in *AKT1-3* occur in 3-5% of human cancers. Although a single hotspot mutation, E17K, is the most common, well characterized activating mutations account for a minority of Akt variants that have been identified in large tumor sequencing studies to date. In order to determine the potential clinical relevance of both common and rare Akt mutations, we expressed a set of over twenty recurrent Akt mutants in three different cell lines and evaluated activation of Akt pathway signaling and effects on growth. We determined their relative sensitivity to allosteric and ATP-competitive Akt inhibitors in clinical development. Most Akt mutants did not activate pathway signaling compared to wild type Akt and did not affect growth properties. In addition, the most common activating Akt mutations, including Akt1 E17K, L52R, and Q79K conferred neither sensitivity nor resistance to Akt inhibitors. Equivocal evidence was found that Akt1 D323H and Akt2 W80C mutants are relatively resistant to the allosteric Akt inhibitor MK-2206, but not an ATP-competitive inhibitor. Our results suggest that the vast majority of rare Akt variants are passenger mutations with no effect on drug sensitivity. The hypothesis that activating Akt mutations predict for Akt inhibitor sensitivity remains to be tested clinically, but is not yet supported by our preclinical data.

## INTRODUCTION

With the recent explosion in knowledge of cancer genetics and widespread availability of tumor whole exome or targeted sequencing for clinical decision-making, large clinical trial efforts are underway to implement personalized or “precision” oncology approaches. In simplest form these approaches rely on a tumor's somatic mutation profile to determine which mutations are drivers of tumor growth and the subset of such mutations which are considered “actionable,” meaning they are thought be drivers and are also potentially targetable by an approved or investigational drug available for use. Actionable mutations in a given gene are used as the basis for selection of an appropriate targeted therapy. Although there are clear examples of the success of this strategy, several practical issues become apparent when one attempts to implement such a program in clinical research or clinical practice. First, tumor sequencing identifies genetic variants of unknown significance—even when they occur in well accepted oncogenes. One cannot assume that all variants in a known oncogene are functionally activating. Passenger mutations may occur even in a known oncogene, and the cancer may not in fact be “addicted” to this mutant gene. Second, not all activating mutations in an oncogene will confer the same susceptibility to targeted inhibitors. For example, a small percentage of B-Raf mutant cancers have non-V600 mutations which have been shown to result in a kinase-dead form of B-Raf that nonetheless activates the MAPK pathway via C-Raf [1]. Such mutants are not sensitive to the B-Raf V600-specific inhibitors vemurafenib and dabrafenib. For these reasons it is imperative to have as much specific information about the functional activity of these mutations as possible to optimally apply precision oncology in the clinic. Although the highest level of evidence is from actual patients who have these mutations and are treated with targeted inhibitors, such information will be difficult to collect for relatively rare variants. Preclinical functional analysis may serve in the interim to provide the best available evidence for matching gene variants to targeted therapies.

**Figure 1:**
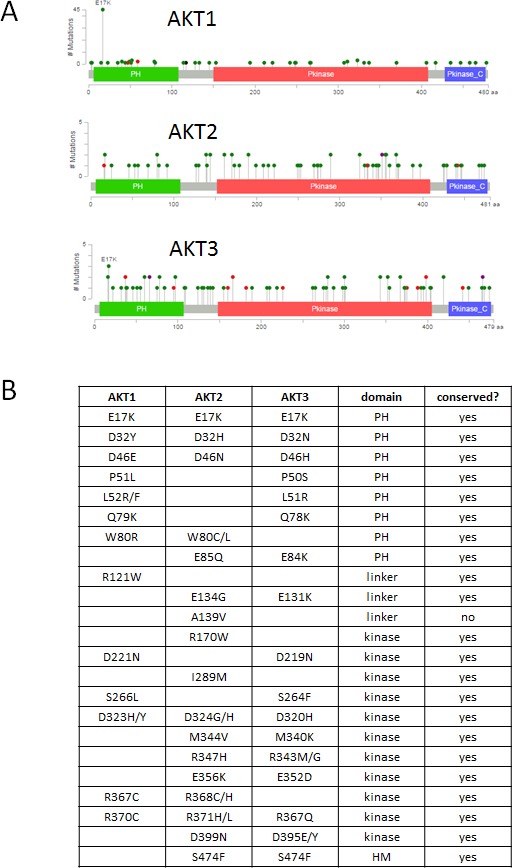
Spectrum of mutations in AKT1, AKT2, and AKT3 **A**. Figure and data derived from cBioPortal website [3]. Pleckstrin homology (PH), kinase, and C-terminal regulatory domains are depicted. Mutations are color coded by type: missense (green), nonsense (red). **B**. Recurrent mutations across *AKT* isoforms analyzed in this study. PH, pleckstrin homology. HM, hydrophobic motif.

Mutations in the highly homologous kinases *AKT1*, *AKT2*, or *AKT3* occur in approximately 3-5% of cancers. A single hotspot mutation G49A:E17K occurs most often in *AKT1*, but the corresponding E17K mutation has also been found in *AKT2* and *AKT3* [2]. E17K accounts for 36% of *AKT1* mutations in cBioPortal but is less prevalent in *AKT2* and *AKT3*. The majority of *AKT1-3* mutations are spread throughout the coding sequence at low frequencies (Figure 1A) [3]. Two major classes of Akt inhibitors are being investigated in clinical trials: allosteric inhibitors and ATP-competitive kinase inhibitors. A number of active or planned clinical trials use *AKT* mutations to determine eligibility for these agents. Previous work from our laboratory and others have identified additional activating mutations in the PH domain of *AKT1-3* [4-6]. Activating kinase domain mutations were also identified by Parikh et al., who proposed that most activating Akt mutations disrupted autoinhibitory PH-kinase domain interactions [4]. Known activating Akt mutations have not been extensively tested for sensitivity to different classes of inhibitors being tested in clinical trials. We therefore sought to determine whether a series of recurrently mutated amino acids across Akt isoforms were functionally activating and whether these mutations conferred either sensitivity or resistance to allosteric or ATP-competitive Akt inhibitors.

## RESULTS

### Functional analysis of pathway signaling by low frequency AKT mutants

We curated a dataset of mutations in *AKT1-3* from COSMIC, TCGA, and individual tumor sequencing studies reported in the literature [3, 7, 8]. We also considered reported mutations in mosaic overgrowth syndromes which frequently involve PI3K pathway genes. Mutations that occurred more than once at the same conserved amino acid residue or homologous residue across Akt isoforms, but which had not already been functionally characterized, were chosen for study (Figure 1B). Full length cDNA of wild type or mutant *AKT1* or *AKT2* were cloned into a retroviral expression vector with an in-frame N-terminal HA epitope tag and infected into several cell lines to generate stable expressing pools of cells. In some cases, multiple variant amino acids at a given residue were evaluated (e.g. Akt2 D32H and D32N, although the former did not express well), whereas in other cases a single variant at a given position was chosen for study (e.g. Akt2 W80C). Although some of the mutants chosen for study occur in *AKT3*, we used only *AKT1* and *AKT2* for our analysis, since all of these *AKT3* mutants had a homologous mutant in *AKT1* or *AKT2*. While there are some data showing isoform-specific functions that differ between Akt1 and Akt2, the signaling pathway is highly conserved, and few functional differences have been described for Akt3 versus Akt2. In order to observe phenotypic similarities across different cellular contexts, we expressed our mutant panel in IL-3-dependent BaF3 murine pro-B cells, Rat1a fibroblasts, and MCF-10A, an EGF-dependent immortalized human breast epithelial cell line. Akt1 L52R and Q79K mutants were not expressed in BaF3, as we and others had previously demonstrated that these mutants activated signaling in multiple cell types and have transforming activity [4, 5].

**Figure 2:**
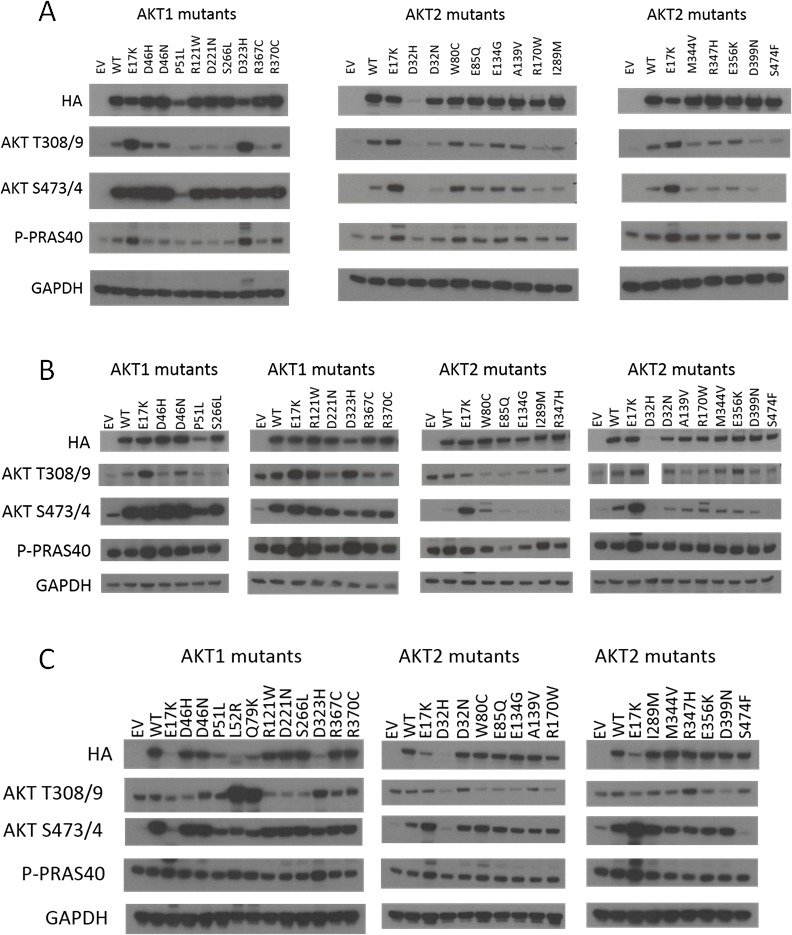
Signaling pathway activation by hotspot and non-hotspot Akt1 and Akt2 mutants Immunoblotting was performed on lysates from: **A**. IL-3-deprived BaF3 cells. **B**. Serum-starved Rat1a cells. Note, Akt T308/9 western shown is from the same gel; however samples were in a different order from the other blots and Akt2 D32H was not run. **C**. MCF-10A cells cultured in the absence of EGF. EV, empty vector control. WT, wild type. Note that all images are cropped to show only the relevant bands.

We first investigated whether these mutations activated Akt and Akt-dependent signaling by western blotting. Known activating mutants of Akt cause increased baseline phosphorylation at the S473/474 and T308/309 residues (of Akt1 and Akt2, respectively), which are essential for full activation of the Akt kinases. This in turn leads to increased phosphorylation of canonical downstream Akt targets, such as PRAS40. As shown in Figure 2, wild type Akt1 or Akt2 overexpression could lead to modest increases in pathway activity in IL-3 deprived BaF3 cells (Figure 2A) or serum-starved Rat1 cells (Figure 2B). The known activating mutants Akt1 E17K, L52R, Q79K, and D323H all demonstrated increased Akt and PRAS40 phosphorylation compared to wild type. None of the other non-hotspot Akt1 mutants showed clear evidence of activation greater than wild type Akt1. These same mutants exhibited similar activity when overexpressed in the breast epithelial cell line MCF-10A; however, the expression levels of the mutants were more variable in this cell line, with lower expression of the most strongly activating mutants (Figure 2C). This may indicate an intolerance of the MCF-10A cells to such high Akt pathway activity, a phenomenon we have previously observed when MCF-10A *PIK3CA* mutant knock-in cells are maintained in high-EGF culture media [9].

Similarly, for Akt2, the E17K mutant clearly activated the pathway, but none of the other mutants tested, with the exception of the PH domain mutant W80C, showed pathway activity above wild type Akt2. Akt2 W80C increased phosphorylation of Akt2 S474 and PRAS40 in BaF3 and of Akt2 S474 alone in Rat1a and MCF-10A, suggesting that this mutation may be weakly activating.

### Transforming activity of Akt mutants

**Figure 3:**
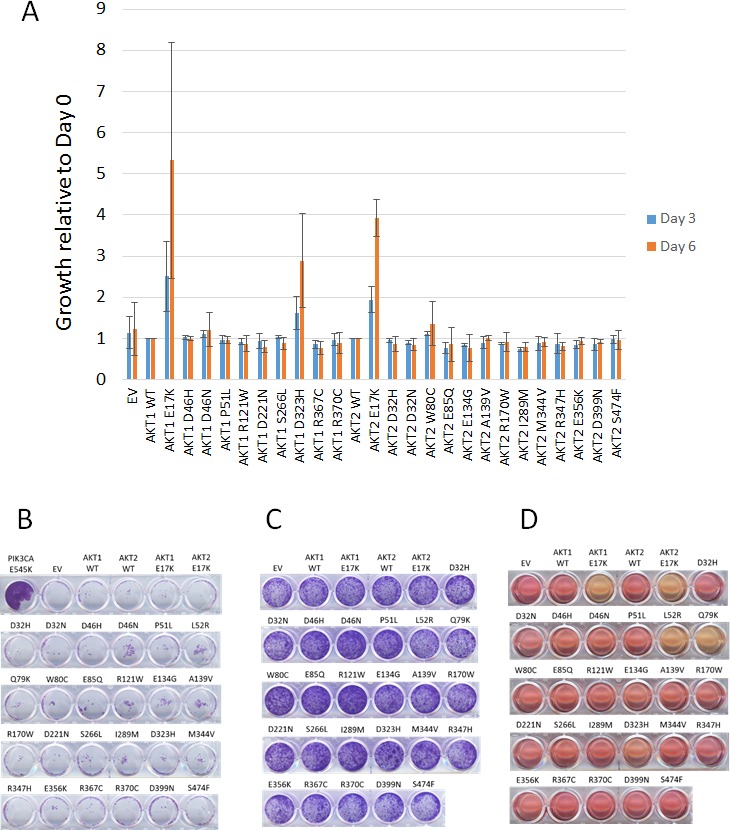
Effects of mutant Akt proteins on growth properties of BaF3, MCF-10A, and Rat1a cells **A**. IL-3 independent growth of BaF3 cells. Fold increase at days 4 and 6 relative to growth of AKT wild type cells. AKT1 mutants are normalized to AKT1 wild type, and AKT2 mutants are normalized to AKT2 wild type. **B**. EGF-independent growth of MCF-10A cells expressing Akt proteins. Crystal violet staining after 14 days of growth is shown. *PIK3CA* E545K knock in MCF-10A cells are shown as a positive control. **C**. Growth of Rat1a cells expressing Akt proteins assessed by crystal violet staining. **D**. Media acidification of confluent Rat1a cells expressing Akt1 E17K, Akt2 E17K, Akt1 L52R, Akt1 Q79K, and Akt1 D323H.

To further characterize the oncogenic properties of these mutants, we tested their ability to maintain survival of BaF3 cells after withdrawal of IL-3. As shown in Figure 3A, only the Akt1 E17K, Akt2 E17K, and Akt1 D323H mutants promoted BaF3 survival compared to wild type Akt proteins. Note that the Akt1 L52R and Q79K mutants were not tested in BaF3, as we and others have previously shown that they have oncogenic properties [4, 5]. We had previously demonstrated that targeted knock-in of the *AKT1* E17K mutation in MCF-10A cells is insufficient to cause EGF independence, but the possibility remained that supraphysiologic expression of Akt mutants might lead to growth factor independence [10]. MCF-10A cells expressing the panel of Akt mutants remained completely dependent on EGF for growth, however, although *PIK3CA* E545K mutant knock-in MCF-10A cells grew without EGF as expected (Figure 3B) [9]. Some groups have reported that overexpression of activated forms of Akt can lead to aberrant morphogenesis phenotypes when MCF-10A cells are cultured in a three dimensional basement membrane matrix (Matrigel), although our MCF-10A *AKT1* E17K knock-in cells resembled wild type MCF-10A cells in this assay [4, 10]. We therefore tested whether our panel of *AKT* mutants would lead to aberrant acinar morphogenesis in Matrigel. Again, none of the mutants, including known activating mutants, was capable of transforming MCF-10A cells in this assay. All acini showed normal morphology (data not shown).

Rat1a cells expressing the mutants grew with similar kinetics to empty vector control and wild type Akt-expressing cells (Figure 3C). However, after becoming confluent, certain Akt mutant-expressing cells were reproducibly observed to rapidly acidify the culture media (Figure 3D), perhaps indicating enhancement of glycolytic metabolism by these mutants.

### Akt mutation effects on sensitivity to Akt inhibitors

Two classes of Akt inhibitors in clinical trials are allosteric inhibitors, such as MK-2206, and ATP-competitive kinase inhibitors, such as GSK690693, AZD5363, and GSK2141795 [11-14]. It remains uncertain to what extent activating *AKT* mutations predict for enhanced sensitivity to such agents. Some reports have suggested that certain mutants such as E17K, L52R, and D323H are more resistant to an allosteric inhibitor, Akt inhibitor VIII, although we observed potent biochemical inhibition by MK-2206 of Akt1 E17K in two knock-in cell line models and equivalent growth inhibition compared to isogenic wild type cells [4, 15, 16]. The Q79K mutant has been shown to respond to MK-2206 *in vitro*, although relative sensitivities of this and other mutants to various inhibitors have not been established [6]. The artificial W80A PH domain mutation has been reported to confer resistance to allosteric Akt inhibitors, including Akt inhibitor VIII and MK-2206 [16-19]. It has also been shown recently that certain kinase-inactive Akt mutants can still modify pathway signaling through PH-dependent, but kinase-independent, mechanisms, potentially affecting drug sensitivity [16]. In theory then, a mutation could affect drug binding or activity, which could allow the mutant Akt protein to maintain Akt signaling despite not itself being an activating mutant. We therefore tested our entire panel of Akt mutants for effects on cellular sensitivity to MK-2206 and GSK690693.

**Figure 4:**
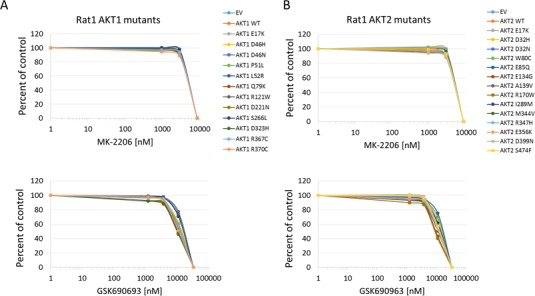
Sensitivity of Rat1a cells expressing Akt mutants to allosteric and kinase inhibitors **A**. Akt1 mutants. Top panel, MK-2206. Bottom panel, GSK690693. **B**. Akt2 mutants. Top panel, MK-2206. Bottom panel, GSK690693. Cell numbers were quantified by Alamar Blue absorbance assay and normalized to DMSO controls. Averages of two experiments are shown.

**Figure 5:**
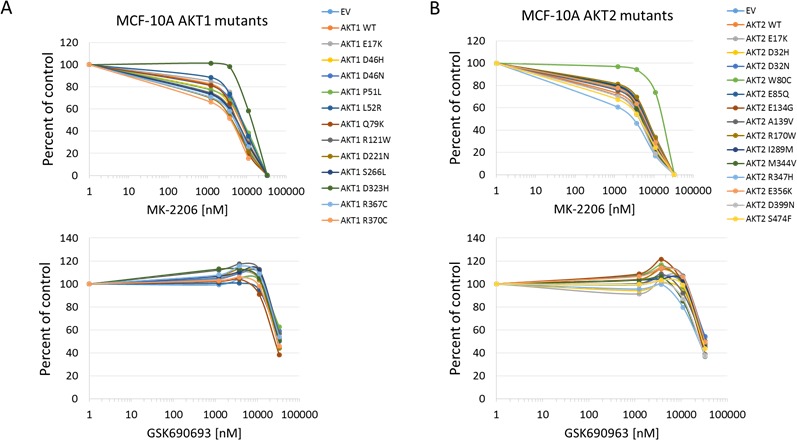
Sensitivity of MCF-10A cells expressing Akt mutants to allosteric and kinase inhibitors **A**. Akt1 mutants. Top panel, MK-2206. Bottom panel, GSK690693. **B**. Akt2 mutants. Top panel, MK-2206. Bottom panel, GSK690693. Cell numbers were quantified by Alamar Blue absorbance assay and normalized to DMSO controls. Averages of two experiments are shown.

**Figure 6:**
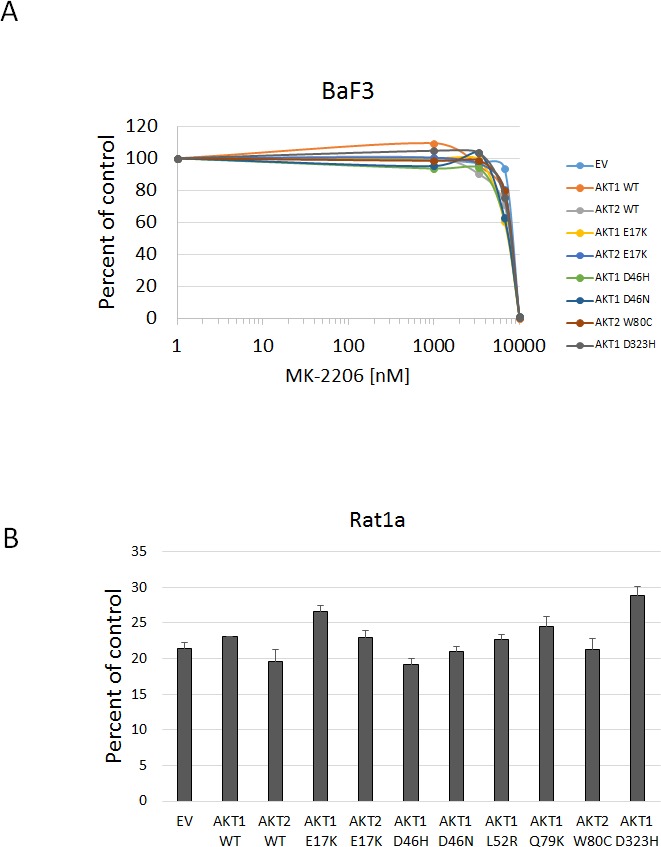
Sensitivity of BaF3 and Rat1a cells expressing Akt mutants to MK-2206 under low growth factor conditions **A**. BaF3 cells stably expressing the indicated mutants and controls were grown in medium containing 0.1 ng/mL IL-3 and treated with MK-2206. Cell numbers were quantified by Alamar Blue absorbance assay and normalized to DMSO controls. Averages of two experiments are shown. **B**. Sensitivity of Rat1a cells expressing Akt mutants grown in low serum to MK-2206. Cells stably expressing the indicated mutants and controls were grown in medium with 0.5% FBS and treated with 1 μM MK-2206. Cell numbers were quantified by Alamar Blue absorbance assay and normalized to DMSO controls. Average and standard deviation of two experiments are shown.

In all cell lines tested, the Akt1 E17K, Akt2 E17K, Akt1 L52R, and Akt1 Q79K mutants showed similar sensitivity to empty vector and wild type Akt-expressing cells to both MK-2206 and GSK690693, indicating that these PH-domain mutants do not confer either sensitivity or resistance to these agents to a significant degree (Figures 4, 5, 6 and Supplementary Figures 1 and 2). Rat1a cells tested in full serum showed no sensitivity to MK-2206 up to 3.3 microM, but then were completely killed at 10 microM, with no separation of the curves, indicating an insignificant degree of variation among the different mutants (Figure 4). A subset of the mutants was re-tested in low serum conditions, and again the therapeutic response profile was similar with 70-80% growth inhibition at 1 microM MK-2206 (Figure 6B). Although Akt1 E17K, Akt1 D46H, and Akt1 D323H differ significantly (*P* < 0.05) from wild type, the observed differences in growth inhibition are small. The only exceptions to this rule were the Akt1 D323H and Akt2 W80C mutants, which were more resistant to MK-2206 when expressed in the MCF-10A cell line (Figure 5). However, the IC50 difference was significant only for W80C after correction for multiple comparisons (*p* < 0.01; Supplementary Figure 1). These two mutants did not cause resistance to MK-2206 in Rat1a or BaF3 cells, however (Figures 4 and 6 and Supplementary Figure 2). Both mutants were as effectively inhibited by GSK690693 as the rest of the mutant panel in both Rat1a and MCF-10A cells (Figures 4 and 5).

## DISCUSSION

Large scale tumor sequencing studies have been successful in identifying the landscape of driver oncogenes in human cancers. Taken to the next level, clinical sequencing of tumors is being used for personalized oncology, with the notion that sequencing can identify the likely drivers in an individual patient's cancer. The secondary assumption is that the presence of such a mutant driver will predict clinical benefit when a drug targeting that driver is used. As we show in this study, there are important caveats to this approach. First, although sequencing is good at identifying tumor drivers in general (i.e., Akt proteins are clearly human oncogenes), clinical sequencing is less robust for identifying the drivers in any particular individual's cancer. Certain oncogenic drivers are clearly more potent, or more addicting than others, and their role may vary depending on the cell of origin, as well as the genetic and epigenetic context of the tumor. Second, the effects of many mutations (especially non-hotspot mutations) in known oncogenes on protein function and drug sensitivity are unknown, and all variants cannot be assumed to be activating and drug-sensitizing.

*AKT* mutations highlight these issues for precision oncology. Akt proteins are clearly oncoproteins, and overexpression of activated Akt isoforms can transform cells. The only true hotspot mutation in *AKT1-3* is E17K, however. Its high frequency (∼15% of all *AKT1-3* mutations in cBioPortal) suggests that it is a bona fide driver, and multiple studies, including this one, have shown that E17K activates the Akt signaling pathway and can transform cells. However, non-E17K mutations will account for the majority of *AKT* mutations identified by clinical tumor exome sequencing, and there has been little functional characterization of these rare mutations. In this study we tested 23 different amino acid residues for which we could find at least two mutation instances in the literature and sequencing databases. Only the E17K mutants of Akt1 and Akt2 and the previously validated Akt1 L52R, Q79K, and D323H mutants showed clear pathway activation compared to wild type Akt, and only these mutants showed oncogenic activity in terms of IL-3 independent survival of BaF3 or transformation of Rat1a [4, 5]. L52R and Q79K mutations represent 1.2% each of *AKT1-3* mutations in cBioPortal, which is only slightly higher than our threshold frequency, representing occurrence at least twice in reported studies (equivalent to ∼0.6%). Mutations in the R370/R371/R367 residues are as prevalent in COSMIC as L52/L51 or D323/D324 (1.2%), but these mutations do not appear to be activating. It is likely that with the accumulation of more sequencing data, we will have a better understanding of the true frequency of these different mutations, and perhaps a clearer distinction between potential drivers and passengers based on frequency of occurrence will emerge. Another caveat is that we did not test additional mutations with only a single reported occurrence. We and others have identified a few activating *AKT1* mutants which have been reported only once, so it remains possible that additional rare activating mutations will be found. However, our study suggests that the vast majority of such rare mutations will be passengers.

It is difficult to rule out the possibility that some of these Akt mutants have a gain of function and role in transformation that is simply too weak to be observed in the assays we performed, or which impacts a phenotype that we did not study. The context-dependent phenotypic effects observed by Parikh et al. for certain Akt mutants suggest that there could be subtle aspects of oncogene activity that vary with biological context. For example, Akt1 K189N increased Akt phosphorylation in MCF-10A but did not promote IL-3 independent growth in BaF3 in the study by Parikh. In our study we saw inconsistent effects of overexpression of some of the mutants. Akt2 W80C increased phosphorylation of Akt2 S474 and PRAS40 in BaF3 and of Akt2 S474 alone in Rat1a and MCF-10A. W80C did not score as positive in our BaF3 proliferation assay, but it is possible that this mutation is weakly activating. It is notable that W80 is adjacent to other PH domain residues activated by mutation, including C77 and Q79, and artificial mutation of T81 also activates Akt [4].

An expectation of precision oncology is that matching targeted therapies with driver mutations will result in improved responses and survival for patients. The common E17K mutant, and the rarer L52R, Q79K, and D323H mutants are all clearly activating and capable of driving transformation. None of these mutants render cells more sensitive to allosteric or ATP-competitive inhibitors compared to wild type Akt, however. In previous work we showed that two different *AKT1* E17K knock-in cell lines were no more sensitive than their isogenic wild type controls to MK-2206, and overexpression of E17K, L52R, and Q79K did not sensitize BaF3, Rat1a, or MCF-10A cells to either MK-2206 or GSK690693 in the current study. A limitation of all of these studies is the possibility that a cell engineered to express a mutation may not become “addicted” to this driver in the same way that an autochthonous driver mutation might. However, we have shown that engineered knock-in of hotspot *PIK3CA* mutations can indeed sensitize these cells to PI3-kinase inhibitors [9, 10].

Another important clinical use of mutation data for precision oncology is to avoid using drugs to which a given mutation confers resistance. Our data suggest that the most common functionally activating Akt mutations, E17K, L52R, and Q79K, are not inherently resistant to MK-2206 or GSK690693. The W80C and D323H mutants did show resistance to MK-2206, but not GSK690693, in a single cell line, MCF-10A. Our results with W80C agree with those of Parikh et al. and Vivanco et al. who found that the artificial W80A mutant was resistant to either Akt inhibitor VIII or MK-2206 in different cell lines [4, 16]. However, we found MK-2206 to be effective against the E17K and L52R mutants in multiple cell lines, whereas Parikh et al. observed little to no growth inhibition in NIH3T3 cells expressing these mutants when treated with Akt inhibitor VIII. It is possible that the different results with MK-2206 reflect structural differences between the two allosteric inhibitors. Recently Davies et al. also found efficacy of MK-2206 against Akt1 E17K mutant cell lines and xenografts[20]. Like Parikh et al., we found the Akt1 D323H mutant to be relatively resistant to an allosteric Akt inhibitor; however, we only observed this effect in a single cell line, and the shift in IC50 was not statistically significant. It is possible that these mutants exhibit a context-dependent resistance to allosteric inhibitors, which may be clarified by further preclinical and eventual clinical data. Until such data are available, caution is suggested in using allosteric inhibitors for W80 and D323 mutations.

In summary, the “long tail” of low frequency, uncharacterized mutations in the *AKT1-3* genes in human tumors is emblematic of challenges facing the field of precision oncology. Our study highlights the possibility that the majority of sequence variants identified in the *AKT* genes will be passenger mutations without clear functional consequences. Such mutations should not be used to prescribe Akt-targeted therapies to patients. For the subset of validated activating Akt mutations, including E17K, the clinical utility of a precision oncology strategy remains unclear. Preclinical data, including ours, has not shown a clear signal of increased sensitivity of Akt mutant cells to Akt inhibitors; however, this hypothesis remains a valid one to be tested in clinical trials. Recent reports have documented responses of a few Akt1 E17K mutant tumors to Akt inhibitors in patient-derived xenografts and phase I clinical trials [20, 21]. Ultimately more data from patients with *AKT* mutant tumors will be required to determine how well *AKT* mutations predict such clinical benefit. Of course, Akt may still be a good therapeutic target even if mutant proteins do not confer *increased* clinical benefit over wild type; the implication would only be that mutations need not be used for selection of Akt-targeted therapies.

## MATERIALS AND METHODS

### Cell lines

Rat1a cells were originally obtained from Dr. Linda Smith-Resar and were grown in DMEM with 4.5g/dL glucose (Mediatech) supplemented with 10% fetal bovine serum (FBS, Hyclone) and penicillin-streptomycin. MCF-10A cells were cultured in DMEM/F12 medium (Mediatech) supplemented with 5% horse serum (Invitrogen), penicillin-streptomycin, 20 ng/mL EGF (Sigma), 0.5 μg/mL hydrocortisone (Sigma), 10 μg/mL insulin (Invitrogen), and 0.1 μg/mL cholera toxin (Sigma). BaF3 cells were obtained from Dr. Patrick Brown (Johns Hopkins University) and maintained in RPMI-1640 medium (Mediatech) supplemented with 10% FBS, penicillin-streptomycin, 50 nM 2-mercaptoethanol, and 0.5 ng/mL IL-3 (R & D Systems). All cells were cultured at 37°C at 5% CO_2_.

### Akt expression constructs

Wild type and mutant versions of human Akt1 and Akt2 coding sequence were cloned by PCR either directly from human cDNA or using PCR mutagenesis to introduce the various AKT mutations. Primer sequences are available on request. The cDNA inserts containing a Kozak consensus sequence and stop codon were cloned into the BamHI and XhoI sites of the pBABEpuroL-HA retroviral vector (derived from pBABEpuroL-PTEN, Addgene plasmid 10785, by excising the PTEN coding sequence). All sequences were verified by sequencing both strands at the Johns Hopkins Synthesis and Sequencing Facility. Constructs were packaged into VSV-G envelope pseudotyped retroviruses by transiently transfecting 293T cells using FuGENE 6 (Promega). Stable pools of cells were selected and maintained in puromycin (Invitrogen).

### Immunoblotting

Whole-cell protein extracts prepared in Laemmli sample buffer were resolved by SDS-PAGE using NuPage 4-12% gels (Invitrogen), transferred to Invitrolon polyvinylidene difluoride membranes (Invitrogen), and probed with primary and horseradish peroxidase–conjugated secondary antibodies. The primary antibodies used in this study are anti-HA (CST #2367), phospho-PRAS40 (T246) (CST#2997), phospho-Akt1 (S473) (D9E) XP (CST #4060), phospho-Akt1 (T308) (CST #2965), phospho-Akt2 (S474) (CST#8599), and GAPDH (CST #5174). Blots were exposed to Kodak XAR film using chemiluminescence for detection (Perkin Elmer).

### Growth assays

BaF3 cells were plated in complete medium without IL-3 in 96-well plates in six replica wells. On days 3 and 6, medium containing 10% Alamar Blue (Invitrogen) was added, and plates were incubated at 37°C for 24 hours before reading absorbance on a plate reader (Omega). MCF-10A cells were washed three times with HBSS, then resuspended in DMEM/F12 supplemented with 1% charcoal dextran-treated FBS, insulin, hydrocortisone, cholera toxin, and penicillin-streptomycin. 2,500 cells per well were plated in 24-well plates, and plates were incubated for two weeks, then fixed and stained with crystal violet. Rat1a cells were seeded in complete growth medium at 5,000 cells per well in 24-well plates, which were fixed and stained with crystal violet after three days.

### Drug assays

Cells were plated at 2,000 cells/well in growth medium in 96 well plates, with six replica wells per drug concentration. The following day, medium was replaced with fresh complete medium containing DMSO vehicle or MK-2206 or GSK690693 (Selleck Chemicals) at the indicated concentrations. On day 6, medium containing 10% Alamar Blue (Invitrogen) was added, and plates were incubated at 37°C for 6 hours before reading absorbance on a plate reader (Omega). Rat1a cells were tested in both complete medium with 10% FBS as well as low (0.5% FBS) serum conditions. MCF-10A cells were grown in complete MCF-10A medium containing 0.2 ng/mL EGF (1% of standard EGF concentration in complete MCF-10A medium). BaF3 cells were tested in medium containing 0.1 ng/mL IL-3.

### Statistics

Cell viability data were plotted using Excel (Microsoft). For IC50 determination, the same data were plotted using GraphPad Prism version 5.0 for Windows (GraphPad Software; www.graphpad.com) and curve-fit using a three parameter non-linear dose-response least squares fit. Statistical comparison of IC50 values was performed using one way ANOVA using Dunnett's multiple comparison test with Akt1 or Akt2 wild type as control. A *p* < 0.05 was chosen as significant.

## SUPPLEMENTARY MATERIAL FIGURES


