# Supplementary material for: Recurrent AKT mutations in human cancers: functional consequences and effects on drug sensitivity
Source: Oncotarget. 2015 Dec 17;7(4):4241–51. doi: 10.18632/oncotarget.6648 (PMC4826202; doi:10.18632/oncotarget.6648)
Supplement: Supplementary file 1 [file oncotarget-07-4241-s001.pdf]

Recurrent *AKT* mutations in human cancers: functional consequences and effects on drug sensitivity

Supplementary Material

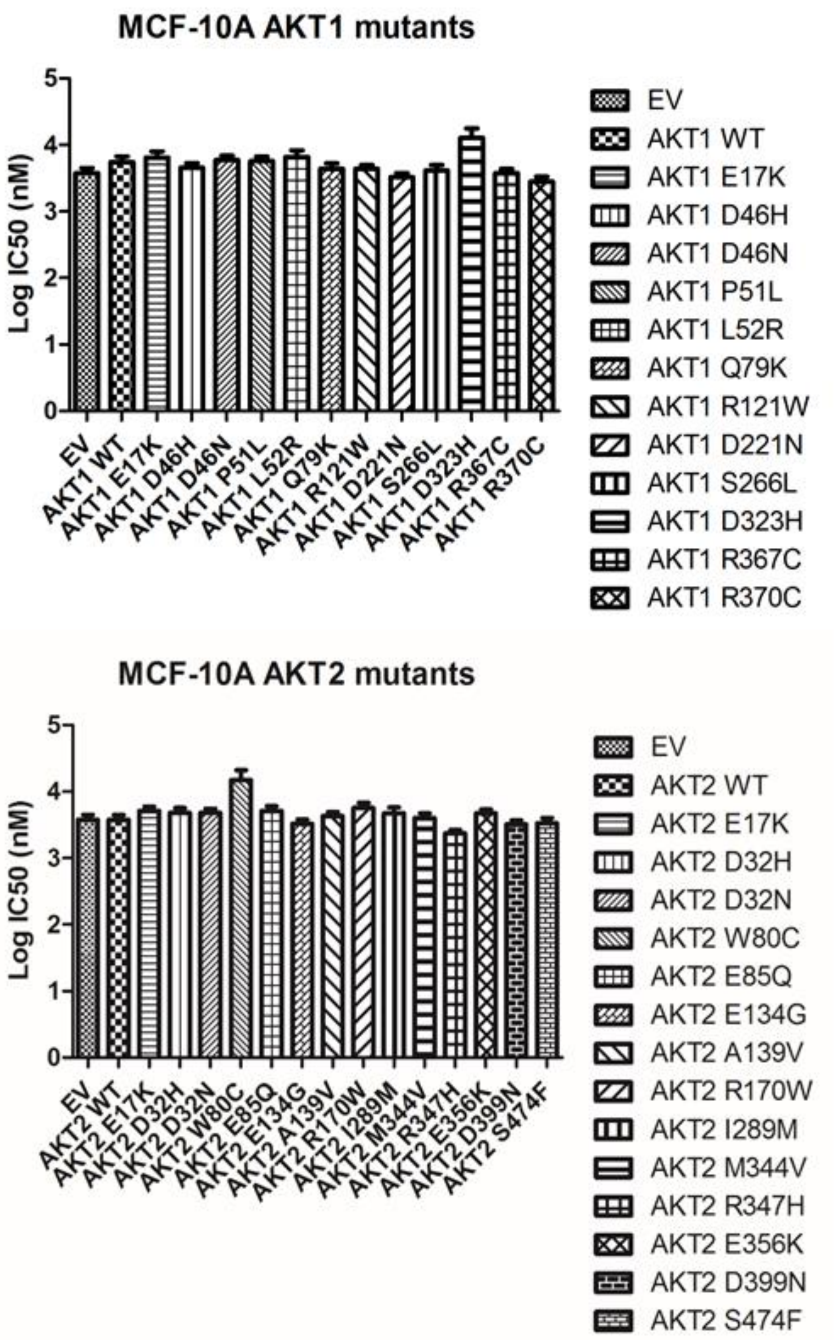

Supplementary Figure 1. MK-2206 Log IC50 concentration (in nM) for MCF-10A cells expressing Akt1 (top) or Akt2 (bottom) mutants. Mean and standard error of the mean are shown. Values were compared to wild type Akt controls using one way ANOVA with Dunnett's multiple comparison test. $P>0.05$ for Akt1 D323H. $P<0.01$ for Akt2 W80C.

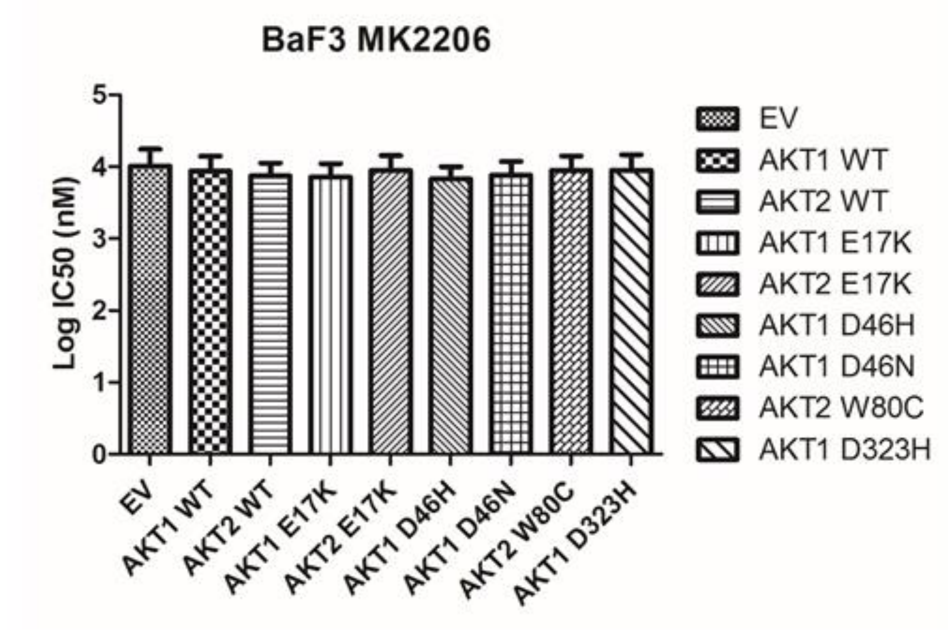

Supplementary Figure 2. MK-2206 Log IC50 concentration (in nM) for BaF3 cells expressing Akt mutants. Mean and standard error of the mean are shown. Values were compared empty vector (EV) controls using one way ANOVA with Dunnett's multiple comparison test. P=ns for all mutants.
